# Correlation between cortical morphology and synaptic-associated proteins levels in poststroke aphasia: a pilot study

**DOI:** 10.3389/fpsyg.2025.1636531

**Published:** 2025-10-16

**Authors:** Yun Cao, Jiaqin Huang, Danli Zhang, Xiaojing Lei, Zhongjian Tan, Jingling Chang

**Affiliations:** ^1^Department of Neurology, Dongzhimen Hospital, Beijing University of Chinese Medicine, Beijing, China; ^2^Department of Traditional Chinese Medicine, Beijing Jishuitan Hospital, Capital Medical University, Beijing, China; ^3^Department of Traditional Chinese Medicine, Beijing Hospital, Beijing, China; ^4^Dongzhimen Hospital, Beijing University of Chinese Medicine, Beijing, China

**Keywords:** poststroke aphasia, cortical morphology, synaptic plasticity, surface-based morphometry (SBM), voxel-based morphometry (VBM)

## Abstract

**Background:**

Poststroke aphasia (PSA) is a leading cause of poststroke disability. The neurobiological mechanisms mediating early recovery, however, remain incompletely characterized—particularly how serum levels of key synaptic proteins correlate with neuroimaging measures of cortical integrity and collectively contribute to language outcomes. This study was therefore designed to examine the relationships between these circulating synaptic markers and structural alterations in the brain of PSA patients to elucidate the potential mechanisms underlying functional recovery.

**Methods:**

A total of 12 PSA patients and 12 healthy controls (HCs) were enrolled in this prospective study. Serum levels of synaptic-associated proteins were measured, and high-resolution 3T structural magnetic resonance imaging (MRI) was performed. Group differences in brain structure were analyzed using voxel-based morphometry (VBM) and surface-based morphometry (SBM). Correlation analysis was conducted among factors with significant group differences.

**Results:**

Compared with HCs, PSA patients had significantly altered serum levels of *α*-SYN, BDNF, TrkB, CREB, and GAP-43. Voxel-wise VBM revealed decreased gray matter volume (GMV) in various regions in PSA patients, including the left postcentral gyrus (PoCG), precuneus (PCUN), superior temporal gyrus (STG), lingual gyrus (LING), inferior parietal gyrus (IPG), middle occipital gyrus (MOG), right superior parietal gyrus (SPG), and superior frontal gyrus (SFG) (uncorrected *p* < 0.001). According to the SBM analysis, comparisons of cortical thickness (CT) revealed significant differences between the groups in the left PCUN, inferior frontal gyrus (IFG), right posterior cingulate gyrus (PCC), etc. Furthermore, patients with PSA presented decreases in sulcal depth (SULC) in the left SFG, right inferior temporal gyrus (ITG), middle temporal gyrus (MTG), and MOG. Correlation analysis revealed significant positive correlations between the repetition score and the CT of the left Precentral Gyrus (PreCG), as well as the SPG.

**Conclusion:**

In summary, patients with PSA exhibit distinct alterations in synaptic protein expression accompanied by widespread gray matter atrophy, marked by reduced GMV, CT, and SULC, particularly in language-related regions. These structural and molecular interrelationships suggest that early recovery involves neuroplastic mechanisms, potentially mediated via synaptic plasticity as well as structural adaptation. Our findings provide novel multidimensional insights into the neurobiological substrate of PSA and highlight promising pathways for future mechanistic and therapeutic research.

## 1 Introduction

Aphasia is an impairment of language production and/or comprehension that is a common and severe consequence of stroke (Boehme et al., 2016; Flowers et al., 2016); approximately one-third of patients experience aphasia during the acute stage, and 61% remain affected at 1 year after onset (Pedersen et al., 2003). Poststroke aphasia (PSA) is also associated with poorer performance in functional recovery (Gialanella et al., 2011), activities of daily living (Paolucci et al., 2000) and emotional well-being after stroke (Thomas et al., 2013), which affects hospital discharge destinations (Gialanella et al., 2009) and the likelihood of a successful return to work. Therefore, there is an urgent need to understand the mechanisms involved in language recovery to develop targeted treatment options for PSA. Many individuals with PSA exhibit some spontaneous recovery of language function, which has a decelerating time course, with the greatest gains occurring early and the slope of change then decreasing (Wilson et al., 2023). Evidence from animal studies shows that a series of neuroplastic changes occur in the brain after acute ischemic injury (Liu et al., 2022). These plasticity processes include the turnover of local synaptic contacts adjacent to the lesion, altered excitability of neuronal circuits adjacent to and connected with the area of damage, and the formation of new functional neuronal connections (Cirillo et al., 2020).

Language, considered the most distinct function of high-level cognition in humans, is controlled by an intricate system involving many brain regions across both hemispheres. In PSA, structural damage triggered by ischemic injury often leads to compensatory reorganization in surviving brain regions, reflected as changes in gray matter volume (GMV), cortical thickness (CT), and surface area (SA) (Cai et al., 2016; Jiang et al., 2017). These morphological adaptations are thought to facilitate functional recovery by compensating for impaired neural circuitry. Although the mechanisms of PSA remain incompletely understood, high-resolution structural MRI—particularly three-dimensional (3D) T1-weighted imaging—has been widely used to investigate such structural alterations. Through techniques such as surface-based morphometry (SBM), which quantifies CT, SA, and cortical volume, and voxel-based morphometry (VBM), which estimates GMV, researchers can detect subtle cortical changes that offer new insights into poststroke neuroplasticity (Shi et al., 2019; Goto et al., 2022). Analysis of these cortical morphological features offers a new perspective for understanding the mechanisms of early brain recovery in patients with PSA. Crucially, since the biomarkers examined in this study are not only localized in gray matter but are also closely associated with its structural recovery (Gupta et al., 2025), structural MRI is a highly suitable imaging technique for investigating these mechanisms.

Language recovery after stroke is supported by both functional reorganization of brain networks and structural changes within these networks, which occur at scales ranging from large-scale circuits to synaptic connections. The macrostructural alterations in gray matter volume and cortical thickness observed by neuroimaging are ultimately underpinned by molecular-level processes, particularly those involving neurotrophic signaling and synaptic plasticity (Pezawas et al., 2004; Tyler and Pozzo-Miller, 2001). Histologically, these imaging parameters represent distinct brain features: gray matter volume is a product of cortical thickness and surface area, each with unique genetic and ontogenetic origins. Critically, the poststroke period triggers a cascade of neuroplastic events that directly influence these structural parameters. Studies of animal models in the acute phase of stroke have shown that growth factors triggered by ischemic events appear within three days and peak between 7 and 14 days (Slevin et al., 2000). Angiogenesis occurs within 4 to 7 days after cerebral ischemia, and collateral circulation is established to supply the ischemic brain area (Hatakeyama et al., 2020). New nerve synapses also rapidly appear around the lesion. This ability of the central nervous system (CNS) to adapt to and reorganize damage in response to cerebrovascular events is known as neuroplasticity. Central to this process is brain-derived neurotrophic factor (BDNF), a key neurotrophin highly expressed in the cerebral cortex (Liu et al., 2020). BDNF facilitates neurogenesis, neuronal differentiation, and synaptic plasticity through its interaction with the TrkB receptor (Lu et al., 2014). Activation of the BDNF/TrkB signaling cascade, which subsequently triggers phosphorylation of the transcription factor CREB (Wang et al., 2018), is fundamental to adaptive reorganization of the brain. Therefore, the cortical atrophy or hypertrophy observed in perilesional areas or distributed language networks—which are closely linked to aphasia recovery outcomes—are not merely gross volumetric changes but also the macroscopic reflections of these underlying molecular and cellular processes. Disentangling the histological underpinnings of these imaging biomarkers, particularly by independently analyzing cortical thickness and area, could provide more specific insights into the therapeutic potential of targeting pathways such as BDNF/TrkB to ameliorate language impairment after stroke. However, the relationship between cortical morphology and synaptic protein levels in PSA remains largely unexplored. Elucidating this link is therefore essential to integrate macroscopic neural alterations with molecular mechanisms for a holistic understanding of this disorder.

The aim of this study was to investigate the potential biological mechanisms underlying alterations in language function in patients with PSA by integrating cortical morphology with the expression of synaptic-associated proteins and to offer potential insights for the early spontaneous recovery of stroke patients.

## 2 Materials and methods

### 2.1 Participants

The present study was approved by the Ethics Committee of Dongzhimen Hospital Affiliated with Beijing University of Chinese Medicine. Between 20 January 2021 and 20 January 2022, twelve PSA patients were enrolled from the Department of Neurology, Dongzhimen Hospital, Beijing University of Chinese Medicine; additionally, twelve healthy controls (HCs) were also recruited. PSA patients meeting all of the following criteria were included in this trial: (1) diagnosed with ischemic stroke via computed tomography or MRI methods at 14 to 30 days after symptom onset; (2) 18 to 80 years of age, the native language being Chinese and right-handed; (3) primary school and above education level with no serious heart, liver, or kidney diseases; (4) clear consciousness and no cognitive impairments; (5) normal language function before stroke onset and dominant language dysfunction after stroke with mild limb dysfunction; (6) specific aphasia syndrome diagnosed as nonfluent aphasia via the Western Aphasia Battery-Chinese version (WAB-C); (7) Boston Diagnostic Aphasia Examination (BDAE) score of 2 to 4; and (8) able to cooperate for the 30-minute MRI examination. The exclusion criteria were as follows: (1) severe hearing impairments, dysopia or dysarthria; (2) language dysfunction caused by congenital or childhood diseases; (3) received pacemaker surgery, coronary intervention, or coronary artery bypass surgery or had other metal products found in the body; (4) history of brain tumor, brain trauma, or neuropsychiatric disorders. As for the inclusion criteria for the HCs group, participants were required to satisfy the following conditions: (1) closely matched age and educational levels with the PSA patient cohort, and (2) right-handedness.

### 2.2 Blood sample collection and analysis

Peripheral blood was collected from each participant. The synaptic-associated proteins include *α*-Synuclein (α-SYN) (ng/mL), Brain-derived Neurotrophic Factor (BDNF) (ng/mL), Tropomyosin receptor kinase B (TrkB) (ng/mL), cAMP response element-binding protein (CREB) (ng/mL), Growth-Associated Protein 43 (GAP-43) (pg/mL), and Glutamate Receptor (GluR) (mg/L).

### 2.3 Behavioral assessments

Before each MRI scan, the severity of language impairment in patients with PSA was assessed by the Chinese version of the WAB. The WAB includes both linguistic subtests, including spontaneous speech, auditory comprehension, repetition, and naming, and nonlinguistic subtests, including subtests for reading, writing, praxis, and construction. The linguistic subtests were analyzed in the current study, and the Aphasia Quotient (AQ) score of the WAB, which reflects the global severity and type of aphasia, was also calculated.

### 2.4 MRI data acquisition

In this study, MRI data were exclusively collected from patients with PSA and HCs. The MRI examinations were conducted using a 3.0 Tesla scanner (Siemens AG, Germany) at Dongzhimen Hospital affiliated with Beijing University of Chinese Medicine. Sagittal structure images were acquired using a magnetization-prepared rapid gradient-echo 3D T1-weighted sequence with the following parameters: repetition time (TR)/echo time (TE) = 1900 ms/2.13 ms, flip angle: 9°, inversion time = 1,100 ms, voxel size: 1.0 × 1.0 × 1.0, resolution: 256 × 256, slice thickness: 1-mm slice thickness without slice gap.

### 2.5 Data preprocessing and MRI indices computation

For each patient, the lesion was manually delineated slice-by-slice on spatially normalized T1-weighted images using MRIcron^1^, under the supervision and verification of an experienced radiologist. The resulting binary lesion masks were used to calculate total lesion volume per subject by counting all non-zero voxels. Each individual lesion mask was then normalized to Montreal Neurological Institute (MNI) space, and a group-level lesion probability map was generated to visualize the spatial distribution of lesions across the cohort.

T1-weighted imaging data preprocessing were carried out via the Statistical Parametric Mapping software (SPM12)^2^ based on MATLAB 2021b (MathWorks Inc., Natick, MA, United States). The VBM and SBM analyses were conducted via the Computational Anatomy Toolbox (CAT12)^3^, which is an extension toolbox of SPM12 software. We used the default settings that are described in detail in the manual of the CAT12 toolbox.^4^ The T1 images were spatially registered to the MNI template. The whole-brain structural data were subsequently segmented into white matter, gray matter and cerebrospinal fluid. Bias correction was performed to remove intensity nonuniformities. Segmented images of the gray matter were preserved to assess the amount of volume changes based on spatial registration, and the modulated images of the gray matter could reflect the tissue volumes for use in VBM analysis. Each subject’s total intracranial volume (TIV) was calculated and used as a covariate for further statistical analyses. The normalized gray matter images were subsequently smoothed via a Gaussian filter (12 mm full-width half-maximum, FWHM). The SBM index calculation steps were as follows: First, surface parameters, which included the sulcus depth, gyrification index, and fractal dimension in addition to the CT, were extracted. Then, the surface data were resampled and smoothed with a 15 mm FWHM Gaussian kernel for cortical thickness and a 20 mm FWHM Gaussian kernel for the other indices. Then, surface values are extracted on the basis of the aparc_2009 atlas.

VBM and SBM analyses were performed using two-sample t-tests in SPM 12. To control for potential confounds, age, sex, TIV, and lesion volume were included as covariates in the statistical model. We first applied Family-Wise Error (FWE) and False Discovery Rate (FDR) corrections at the voxel level. As no clusters survived these stringent corrections, we subsequently employed an exploratory analysis strategy using an uncorrected voxel-level threshold of *p* < 0.001 in combination with a minimum cluster-extent threshold to control for false positives to some extent.

### 2.6 Statistical analysis

Statistical analysis of demographic and clinical data was performed using SPSS software (IBM SPSS Inc., Chicago, IL, USA) and GraphPad Prism 9.3.0. Normally distributed continuous data were presented as mean ± standard deviation, while categorical variables were reported as absolute frequency and percentages. For each continuous variable, the assumption of normality distribution was checked by means of Q-Q plots and Shapiro-Wilks test. For skewed continuous variables, medians along with interquartile range (i.e., IQR, first-third quartiles) were reported instead of means and non-parametric tests were performed. Two-sample t-test, Mann–Whitney U-test and Fisher’s exact test were employed to assess differences between groups for demographic information, clinical data, and laboratory data, depending on the type of data. *p* < 0.05 was considered statistically significant. Pearson correlation was applied for the normally distributed data and the Spearman correlation was applied for the non-normally distributed data. Correlation networks were constructed based on the significant correlations between factors across all subjects. For the correlation analyses, FDR correction was applied to correct for multiple comparisons.

### 2.7 Ethical considerations

This study was conducted in accordance with the Declaration of Helsinki. We confirm that all methods were performed in accordance with the relevant guidelines and regulations. In accordance to the national law on the protection of individuals taking part in biomedical research, participants were informed by their doctors that their biological samples could be used for research purposes and they have been given and signed written consent from before recruited into our study. Ethical approval was approved by the Ethics Committee of Dongzhimen Hospital Affiliated with Beijing University of Chinese Medicine (approval no., 2020DZMEC-092-02).

## 3 Results

### 3.1 Demographic and clinical information of the participants

**Figure 1:**
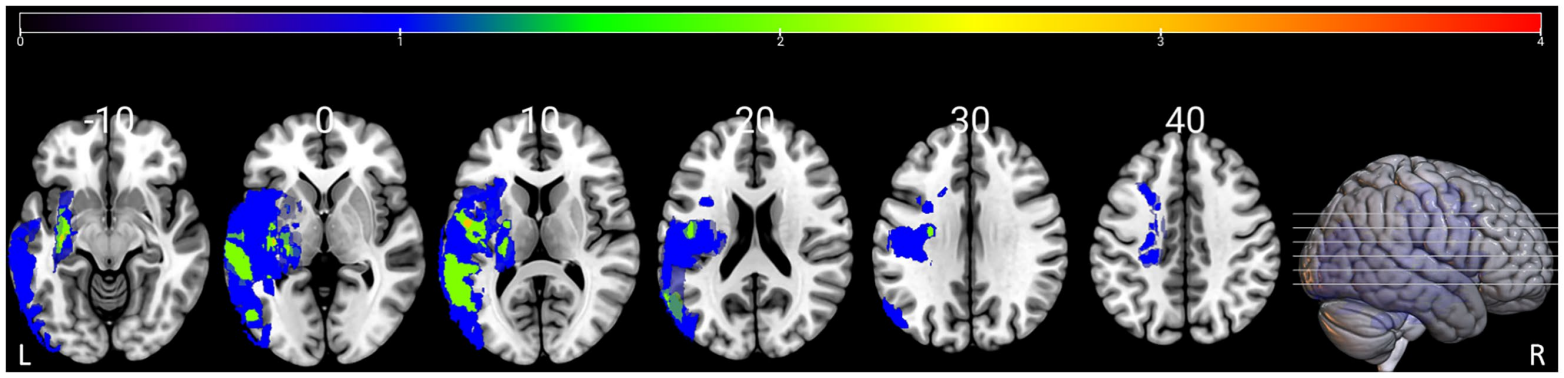
Lesion overlap map for the 12 patients with PSA in MNI space. Color bar represents the number of patients with overlapping lesions at certain locations. The numbers refer to the MNI coordinate space in the z plane. L = left, R = right.

There were no significant inter-group differences in terms of age, sex or education level (all *p* > 0.05). Table 1 shows the demographic information, language assessment scores of the participants. The lesion overlap map for all patients with PSA is presented in Figure 1.

**Table 1:** Demographics, language assessments between PSA and HCs.

Characteristics	PSA (*n* = 12)	HC (*n* = 12)
Demographics		
Age (y)	60.33 ± 10.03	57.08 ± 7.51
Gender (M/F)	8/4	6/6
Education (y)	11.42 ± 2.28	12.08 ± 3.03
Duration (day)	24.67 ± 4.60	NA
Language assessment		
WAB-AQ	63.80 (26.68,77.60)	NA
WAB-spontaneous speech	9.75 ± 4.20	NA
WAB-auditory comprehension	164.50 (124.25,199.50)	NA
WAB-repetition	49.83 ± 39.39	NA
WAB-naming	52.83 ± 34.27	NA

PSA, poststroke aphasia; HC, healthy control; M, male; F, female; WAB, western aphasia battery; AQ, aphasia quotient; y, year; NA, not available.

### 3.2 Differences in synaptic-associated protein expression between groups

**Figure 2:**
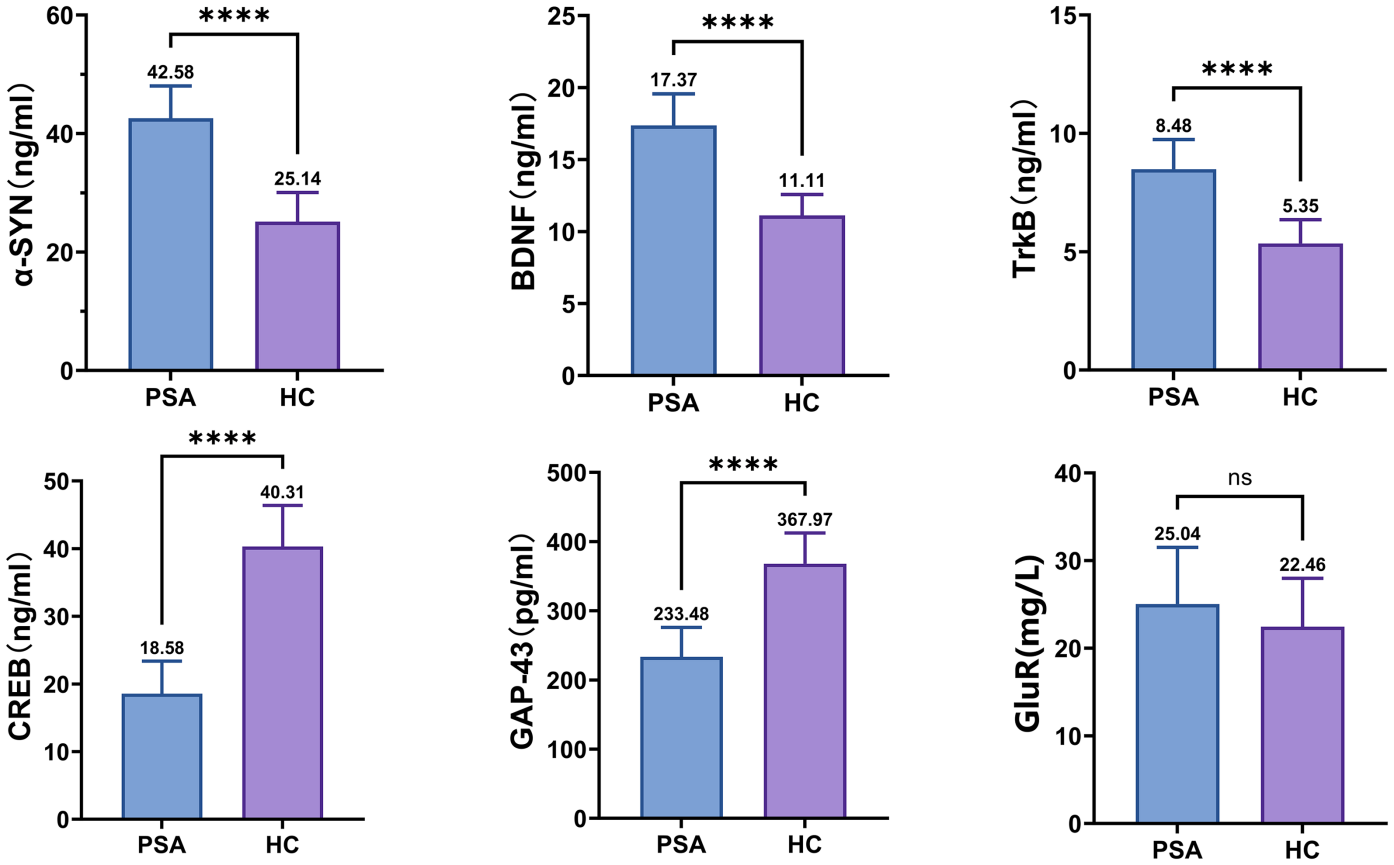
Differences in synaptic-associated protein expression between groups. *α*-SYN, α-Synuclein; BDNF, Brain-derived Neurotrophic Factor; TrkB, Tropomyosin receptor kinase B; CREB, cAMP response element-binding protein; GAP-43, Growth-Associated Protein 43; GluR, Glutamate Receptor; NS, no significance. The mean value is indicated atop each bar. *p < 0.05, **p < 0.01, ***p < 0.001, ****p < 0.0001.

In terms of synaptic-associated proteins, the PSA patients showed higher *α*-SYN, BDNF, and TrkB than HCs, while the levels of CREB and GAP-43 exhibit a lower magnitude in comparison to the HC group (all *p* < 0.0001) (Figure 2).

### 3.3 Differential brain regions in the VBM indices between groups

**Figure 3:**
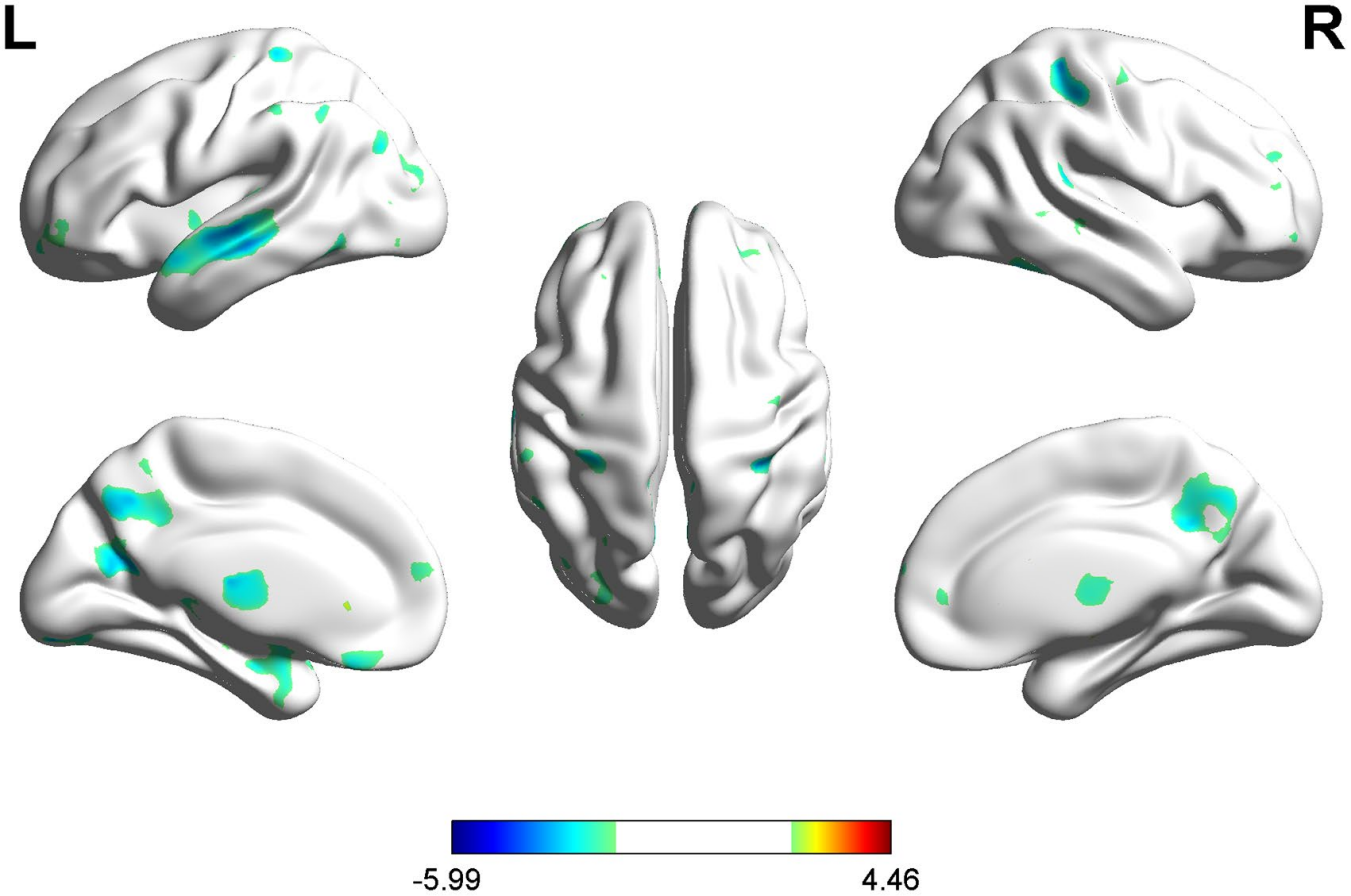
VBM analysis showed reduced GMV in PSA patients compared to HCs (uncorrected cluster-level *p* < 0.001, cluster size≥30 voxels). L = left, R = right.

Voxel-wise VBM analysis revealed reduced GMV in multiple regions in PSA patients, encompassing the left PoCG, PCUN, STG, LING, IPG, MOG, and the right SPG and SFG (uncorrected *p* < 0.001) (Table 2; Figure 3).

**Table 2:** Brain regions with differences in GMV between PSA patients and HCs.

Comparisons	Hemisphere	Brain regions	Size (voxels)	Peak coordinates			*t*
				*x*	*y*	*z*	
PSA < HC	L	PoCG	637	−39	−38	68	4.82
	L	PCUN	244	−17	−60	39	4.74
	L	STG	444	−53	−11	−5	4.64
	L	LING	38	−15	−89	−11	4.2
	L	IPG	108	−3	−60	39	3.88
	R	SPG	86	18	−66	−72	3.86
	L	MOG	107	33	−101	−35	3.82
	R	SFG	56	17	18	−45	3.72

Between-group results were thresholded at an uncorrected cluster-level threshold of *p* < 0.001, with a minimum cluster size of 30 voxels. R, right hemisphere; L, left hemisphere; HC, healthy control; PSA, poststroke aphasia; PoCG, Postcentral Gyrus; PCUN, Precuneus; STG, Superior Temporal Gyrus; LING, Lingual Gyrus; IPG, Inferior Parietal Gyrus; SPG, Superior Parietal Gyrus; MOG, Middle Occipital Gyrus; SFG, Superior Frontal Gyrus.

### 3.4 Differential brain regions in the SBM indices between groups

**Figure 4:**
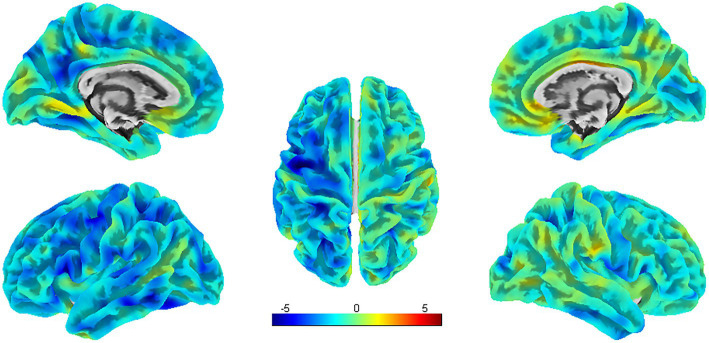
SBM analysis showed significant differences in CT between PSA patients and HCs (uncorrected cluster-level *p* < 0.001, cluster size≥30 voxels). L = left, R = right.

According to the SBM analysis, there were no statistically significant differences between the patients with PSA and HCs regarding the cerebral cortex parameters of the cortical surface area (CSA) and CV. However, a comparison of the CT parameters revealed significant differences between the groups in the left PCUN, PreCG, IFG, and the right PCC, etc. (uncorrected *p* < 0.001) (Table 3; Figure 4).

**Table 3:** Brain regions with differences in CT between PSA patients and HCs.

Comparisons	Hemisphere	Brain regions	Size (voxels)	Peak coordinates			*t*
				*x*	*y*	*z*	
HC > PSA	L	PCUN	258	−47	−6	48	6.17
	L	IFG	168	−47	17	8	5.02
	L	FFG	38	−44	−61	−17	4.99
	L	PoCG	140	−29	−37	54	4.93
	L	MTG	38	−56	−47	−12	4.9
	L	IPL	52	−35	−47	45	4.47
	L	PHG	74	−31	−51	−11	4.32
	L	SPG	56	−31	−55	54	4.11
	L	MFG	37	−33	18	50	4.08
	R	INS	43	40	15	8	3.98
	L	STG	33	−48	−25	8	3.8
	L	PreCG	258	−47	−6	48	6.17
PSA > HC	R	PCC	41	4	−16	29	2.72

Between-group results were thresholded at an uncorrected cluster-level threshold of P < 0.001, with a minimum cluster size of 30 voxels. R, right hemisphere; L, left hemisphere; HC, healthy control; PSA, poststroke aphasia; PCUN, Precuneus gyrus; IFG, Inferior Frontal Gyrus; FFG, Fusiform Gyrus; PoCG, Postcentral Gyrus; MTG, Middle Temporal Gyrus; IPL, Inferior Parietal Lobule; PHG, Parahippocampa Gyrus; SPG, Superior Parietal Gyrus; MFG, Middle Frontal Gyrus; INS, Insula; STG, Superior Temporal Gyrus; PreCG, Precentral Gyrus; PCC, Posterior Cingulate Gyrus.

**Figure 5:**
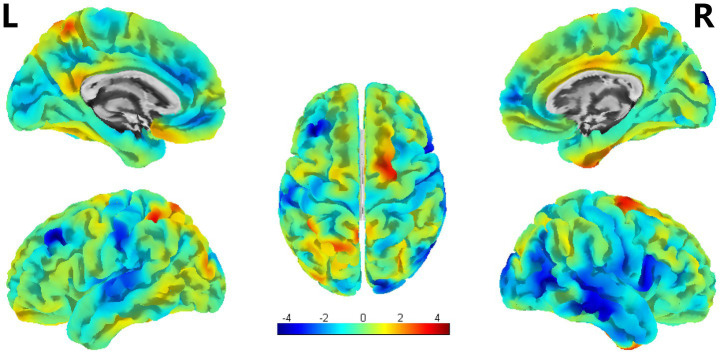
SBM analysis showed reduced SULC in PSA patients compared to HCs (uncorrected cluster-level *p* < 0.001, cluster size≥20 voxels). L = left, R = right.

Compared with HCs, PSA patients presented decreases in SULC in the left SFG, and right ITG, MTG, and MOG; simultaneously (uncorrected *p* < 0.001) (Table 4; Figure 5).

**Table 4:** Brain regions with differences in SULC between PSA patients and HCs.

Comparisons	Hemisphere	Brain regions	Size (voxels)	Peak coordinates			*t*
				*x*	*y*	*z*	
PSA < HC	L	SFG	24	−26	15	54	4.63
	R	ITG	71	54	−21	−22	4.11
	R	MTG	37	56	−28	−5	3.77
	R	MOG	44	47	−65	−10	3.76

Between-group results were thresholded at an uncorrected cluster-level threshold of *p* < 0.001, with a minimum cluster size of 20 voxels. R, right hemisphere; L, left hemisphere; HC, healthy control; PSA, poststroke aphasia; SFG, Superior Frontal Gyrus; ITG, Inferior Temporal Gyrus; MTG, Middle Temporal Gyrus; MOG, Middle Occipital Gyrus.

### 3.5 Associations among language function, synaptic-associated protein levels, and structure MRI indices in the PSA Group

**Figure 6:**
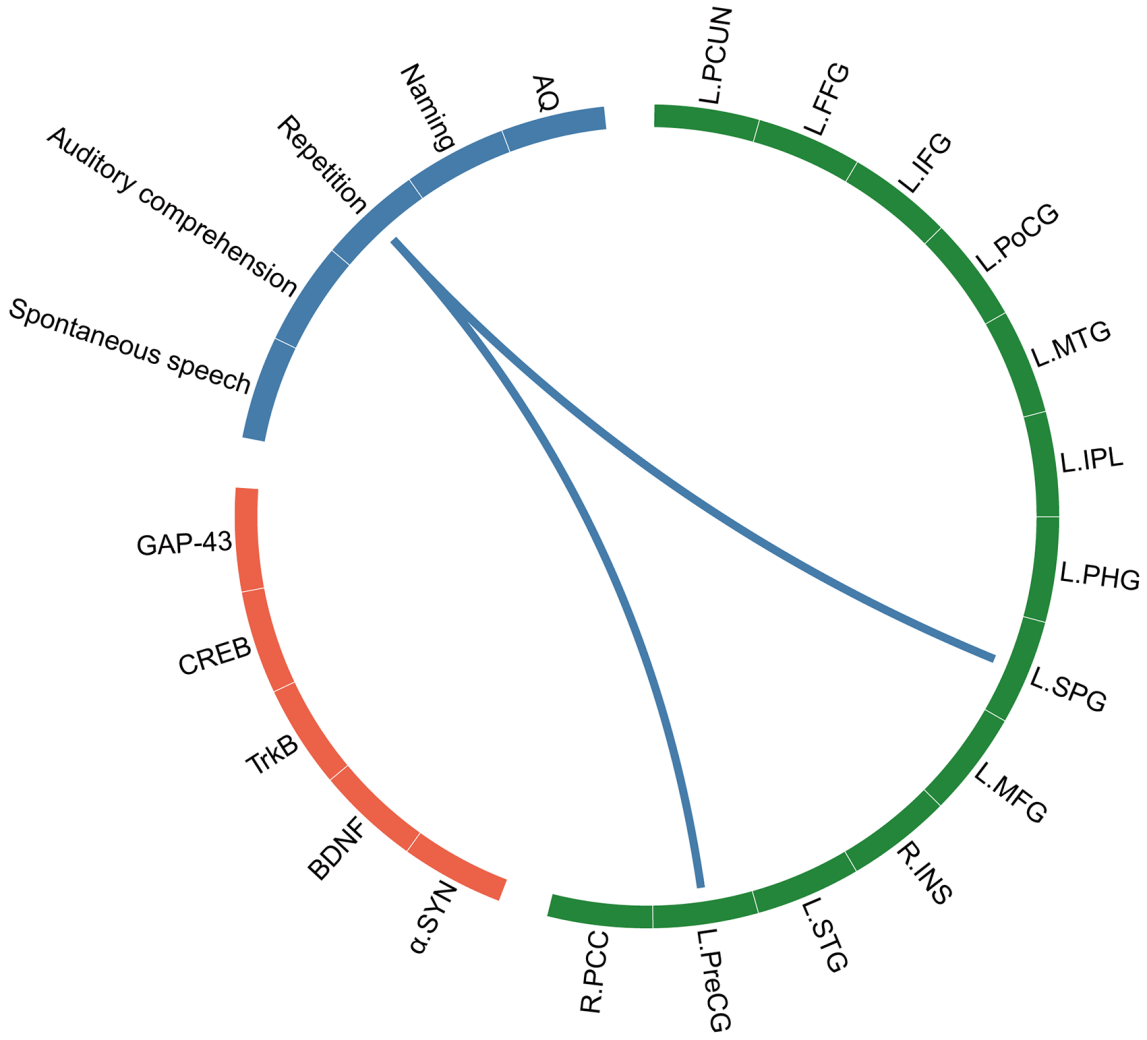
The correlation network of SBM indices (CT), synaptic-associated protein levels and language assessments. The green block represents brain regions identified by SBM analysis, the orange block represents synaptic-associated proteins, and the blue block represents language function.

A correlation network of structural brain characteristics, synaptic-associated protein levels and language function was constructed with the factors that were significantly correlated. Significant correlation was defined as ∣r∣ > 0.3 and *p* < 0.05 after FDR correction (Figure 6). The resulting network revealed that CT derived from SBM were significantly correlated with language function scores, suggesting an association between brain structure and behavior. However, in contrast to our initial hypothesis, neither VBM nor SBM metrics demonstrated statistically significant correlations with synaptic-associated protein levels after FDR correction. To explore potential relationships that did not survive strict multiple comparisons correction, we conducted additional exploratory analyses using a nominal significance threshold (*p* < 0.05, uncorrected). These analyses revealed patterns of association between certain structural brain characteristics, synaptic plasticity markers and language function that, while not surviving FDR correction, may warrant further investigation in larger cohorts. These results are provided in the Supplementary Figure S1.

## 4 Discussion

In this exploratory pilot study—which is among the first to investigate the relationship between serum synaptic biomarkers and macroscopic neuroimaging parameters in patients with PSA—we aimed to elucidate the neurobiological mechanisms underlying early functional recovery. We employed a prospective design including 12 individuals with PSA and 12 matched healthy controls, combining quantitative analysis of circulating synaptic proteins with high-resolution structural MRI. Group comparisons were conducted using both VBM and SBM to identify differences in gray matter volume, cortical thickness, and sulcal depth. Correlation analyses were performed to examine associations among structural brain characteristics, levels of synaptic-associated proteins, and language function. Our results provide initial evidence linking molecular changes in synaptic proteins to structural brain reorganization, offering new insights into the potential mechanisms driving recovery in patients with PSA.

Compared with HCs, PSA patients presented higher *α*-SYN, BDNF, and TrkB levels, whereas the levels of CREB and GAP-43 were lower than those in HCs. After acute stroke, the cascade of primary and secondary neuronal injuries begins with the activation of the endogenous defense pathway. The activation of the endogenous defense mechanism is based on gene expression and protein synthesis, which aid in neuronal restoration via the synthesis and migration of neurotrophic factors or neurotrophic-like molecules (Mureșanu et al., 2022; Wang et al., 2022; Sekerdag et al., 2018). Although neuroinflammation affects several BDNF-related signaling pathways (Lima Giacobbo et al., 2019), its relationship with serum BDNF/TrkB levels in PSA remains largely unknown. Thus, the elevated BDNF and TrkB levels in the early stage of stroke in our study may constitute an adaptive and neuroprotective strategy in response to various types of neuronal insult, which can also be found in diseases associated with cognitive dysfunction (Faria et al., 2014; Ng et al., 2021). Importantly, alterations in synaptic-associated protein levels may be structurally supported by the widespread gray matter atrophy observed in language-related regions, as evidenced by reduced CT. To explore the potential relationships among the variables, we conducted additional exploratory analyses using an uncorrected statistical threshold. Our analyses revealed that the CT of the left FFG, IFG, and IPL were positively correlated with the expression of synaptic-associated proteins. Histologically, the CT is defined by the number of neurons and their connections within the column (Rakic, 1995). The IFG plays a crucial role in language processing and expression, which is widely believed to be closely related to various aspects of language, including auditory and speech processing, reading and writing, and language generation and comprehension (Hallam et al., 2018; Liakakis et al., 2011). Here, we also revealed that the CT of the left IFG was positively correlated with the level of TrkB, which provides a biological link between peripheral synaptic-associated proteins and brain structures in PSA patients. Similar findings were observed for the correlations of *α*-SYN and GAP-43 with the CT of the left FFG and IPL.

The association between specific synaptic protein levels and morphological changes suggests that molecular responses are closely linked to macroscopic cortical reorganization, and together they may influence aphasia recovery outcomes. Notably, it is crucial to interpret these findings with caution, as they indicate association rather than causation. This limitation stems mainly from methodological constraints: human studies in this field are predominantly cross-sectional, providing only a snapshot in time, and it is not feasible to directly sample living brain tissue for detailed molecular analysis. Consequently, the observed relationship may not be direct, but rather driven by a common underlying factor influencing both measures. For example, Polyakova et al. (2020) reported a positive correlation between serum BDNF levels and GMV in individuals with minor depression, although this relationship was both region- and condition-dependent. Similarly, Wu et al. (2023) linked elevated plasma GDNF to reduced GMV in a specific brain region in major depression patients, suggesting a potential neuroprotective or reparative role for GDNF in this disorder. Therefore, although the aforementioned studies offer valuable insights into the relationship between synaptic-associated proteins and structural brain changes, longitudinal and mechanistic research is necessary to establish causality and clarify the directional influence of neuromolecular mechanisms on cerebral reorganization during aphasia recovery.

The results of the present study revealed statistically differences in CT values between PSA patients and HCs in the left PCUN, PreCG, PoCG, and right PCC. Further correlation analysis revealed that the CT of several brain regions were positively correlated with language function; in particular, the CT of the left PreCG and SPG were positively correlated with the language function of repetition. The PreCG is functionally located between the dorsal hand and ventral orofacial cortical representations and exhibits unique sensorimotor and multisensory functions relevant for speech processing, which include motor control of the larynx, auditory processing, reading, and writing. Damage to the PreCG may result in dysarthria, resulting in difficulties with the motor act of speaking and articulating words clearly (Pulvermüller et al., 2006). Accurate repetition relies fundamentally on motor precision; it demands the exquisitely coordinated activation of articulatory muscles to reproduce auditory stimuli, a process orchestrated by the precentral gyrus (Liu et al., 2025). Consequently, damage to this region directly disrupts the motor execution essential for precise repetition. In addition to CT alterations, GMV changes also contribute to the neural architecture of PSA. We found reduced GMV in the left PoCG, PCUN, LING, MOG, and right SPG and SFG. Further correlation analysis revealed that the GMV of the left LING was directly positively correlated with the repetition score. The lingual gyrus is an important region in the brain that contributes to our ability to process and understand language, particularly in relation to visual aspects of language processing. Language understanding is integral to the process of repetition; the former involves interpreting and extracting meaning from text or speech, whereas the latter serves as a tool to reinforce that understanding and ensure clarity. Therefore, language comprehension difficulties can directly impact an individual’s ability to repeat accurately. Furthermore, a positive correlation was also found between the GMV of the left MOG and the naming score. The MOG is part of the occipital lobe located in the posterior region of the brain, specifically within the visual cortex. Research has shown that the MOG does play a role in reading and word recognition processes. Notably, GMV reductions were also observed in the right SPG and SFG. The role of the right hemisphere (RH) in recovery from PSA remains incompletely understood; however, it is widely theorized that RH regions are recruited to support language functions following left hemisphere (LH) damage, primarily through potentiating the right-hemisphere network homologous to the language network—along with other networks that previously supported language to a lesser degree—and by modulating the connection strength between nodes of the RH language network and undamaged nodes in the LH. Although previous studies have reported a compensatory increase in RH GMV during later stages of PSA recovery (Forkel et al., 2014; Lukic et al., 2017), our results demonstrated significant GMV reductions in the RH during the subacute phase (14–30 days post-stroke). This discrepancy may be attributed to differences in the timing of assessment, as prior research often involved patients evaluated more than 6 months post-stroke, whereas the focus of our study was the early recovery period. The subacute period in PSA is widely recognized as a critical interval of enhanced neuroplasticity (Yan et al., 2025), often termed a “window of opportunity” (Wang et al., 2017). During this phase, spontaneous biological recovery processes are most active and interact with experience-dependent plasticity (Li et al., 2024; Grefkes and Fink, 2020). This temporal distinction is essential for interpreting the neuroimaging and synaptic-associated protein changes observed in our study. Despite the absence of a statistically significant direct correlation between RH GMV and language performance in our cohort, both the SPG and SFG are believed to contribute to language recovery through their roles in domain-general cognitive processes. The SPG, for instance, facilitates attentional control and working memory—functions essential for language processing—while the SFG supports aspects of syntax, semantics, speech production, and discourse comprehension. Thus, these right-hemispheric changes may reflect adaptive neuroplasticity involved in the formation of a distributed, bilateral network that supports functional compensation throughout aphasia recovery, even in the absence of straightforward structure–function correlations. Language recovery after stroke is a nonlinear process, with differences in recovery processes and patterns associated with the neuroplasticity of the brain. Synaptic plasticity is a form of neuroplasticity. Synaptogenesis results in new connections that may involve either unmasking previously latent pathways or the formation of new pathways (Cramer, 2008; Kwakkel et al., 2004). Neurons within the gray matter form connections with other neurons through synapses, creating complex neural networks. Therefore, the close relationship between gray matter and synapses enables efficient transmission and processing of information in the brain, allowing for various complex cognitive functions.

There are several limitations of the present study. First, the relatively small sample size of enrolled PSA patients limits the statistical power and generalizability of our findings, and precludes more nuanced subanalyses that might elucidate the precise relationships between synaptic protein levels and neurostructural changes. Future studies with larger cohorts are necessary to validate and extend these preliminary observations. Second, although healthy controls were included, the absence of a control group consisting of stroke patients without aphasia limits our ability to distinguish effects specific to PSA from those attributable to stroke in general. Third, the cross-sectional design and lack of longitudinal follow-up preclude insight into how synaptic-associated protein levels and morphometric measures evolve throughout the recovery process. Fourth, the evidence we provide here is correlational and by no means causative. However, our project was intended as a pilot study and is the first, to the best of our knowledge, to examine potential correlations between serum levels of synaptic-associated protein and changes in the brain structure of patients with PSA.

## 5 Conclusion

In summary, this study demonstrated that patients with early poststroke aphasia exhibit distinct alterations in serum levels of synaptic-associated proteins—including elevated *α*-SYN, BDNF, and TrkB alongside reductions in CREB and GAP-43—accompanied by widespread gray matter structural decline across language-related regions, as reflected by reduced GMV, CT, and SULC. These findings suggest that early spontaneous recovery may involve synaptic plasticity and structural adaptation. Despite its preliminary nature, this study offers novel insights into the neurobiological interplay between structure and function in aphasia recovery.

## Data Availability

The raw data supporting the conclusions of this article will be made available by the authors, without undue reservation.

## Footnotes

^1^ http://www.cabiatl.com/mricro/mricron/index.html

^2^ http://www.fil.ion.ucl.ac.uk/spm/software/spm12/

^3^ http://dbm.neuro.uni-jena.de/cat/

^4^ http://dbm.neuro.uni-jena.de/cat12/CAT12-Manual.pdf

## Glossary

AQ: Aphasia Quotient
BDNF: Brain-derived Neurotrophic Factor
CNS: central nervous system
CREB: cAMP response element-binding protein
CSA: Cortical surface area
CT: Cortical thickness
CV: Cortical volume
FDR: False discovery rate
FFG: Fusiform Gyrus
FWE: Family-wise error
FWHM: Full-width half-maximum
GAP-43: Growth-Associated Protein 43
GluR: Glutamate Receptor
GMV: Gray matter volume
HC: Healthy control
Hipp: Hippocapus
IFG: Inferior Frontal Gyrus
INS: Insula
IPL: Inferior Parietal Lobule
ITG: Inferior Temporal Gyrus
LING: Lingual Gyrus
MFG: Middle Frontal Gyrus
MOG: Middle Occipital Gyrus
MRI: Magnetic Resonance Imaging
MTG: Middle Temporal Gyrus
PCC: Posterior cingulate
PCUN: Precuneus
PHG: Parahippocampa Gyrus
PoCG: Postcentral Gyrus
PreCG: Precentral Gyrus
PSA: Poststroke aphasia
SA: Surface area
SBM: Surface-based morphometry
SFG: Superior Frontal Gyrus
SPG: Superior Parietal Gyrus
STG: Superior Temporal Gyrus
SULC: Sulcal depth
TIV: Total intracranial volume
TrkB: Tropomyosin receptor kinase B
VBM: Voxel-based morphometry
WAB: Western Aphasia Battery
α-SYN: α-Synuclein
