# Supplementary material for: Correlation between cortical morphology and synaptic-associated proteins levels in poststroke aphasia: a pilot study
Source: Front Psychol. 2025 Oct 16;16:1636531. doi: 10.3389/fpsyg.2025.1636531 (PMC12571610; doi:10.3389/fpsyg.2025.1636531)
Supplement: Supplementary file 1 [file Data_Sheet_1.docx]

**Supplementary Materials**

***3.5. Correlations among language function,*** ***synaptic-associated protein levels and structure MRI indices in the PSA group***

The correlation networks depicting the relationships among structural brain characteristics, synaptic-associated protein levels, and language assessments were constructed based on factors showing nominal significance, defined as $|r|$ > 0.5 and *P* < 0.05 (**Figure S1**). This exploratory analysis identified multiple interrelated associations, indicating potential underlying links that merit further investigation. However, as these correlations did not survive correction for multiple comparisons, they should be interpreted with caution and considered hypothesis-generating rather than conclusive.


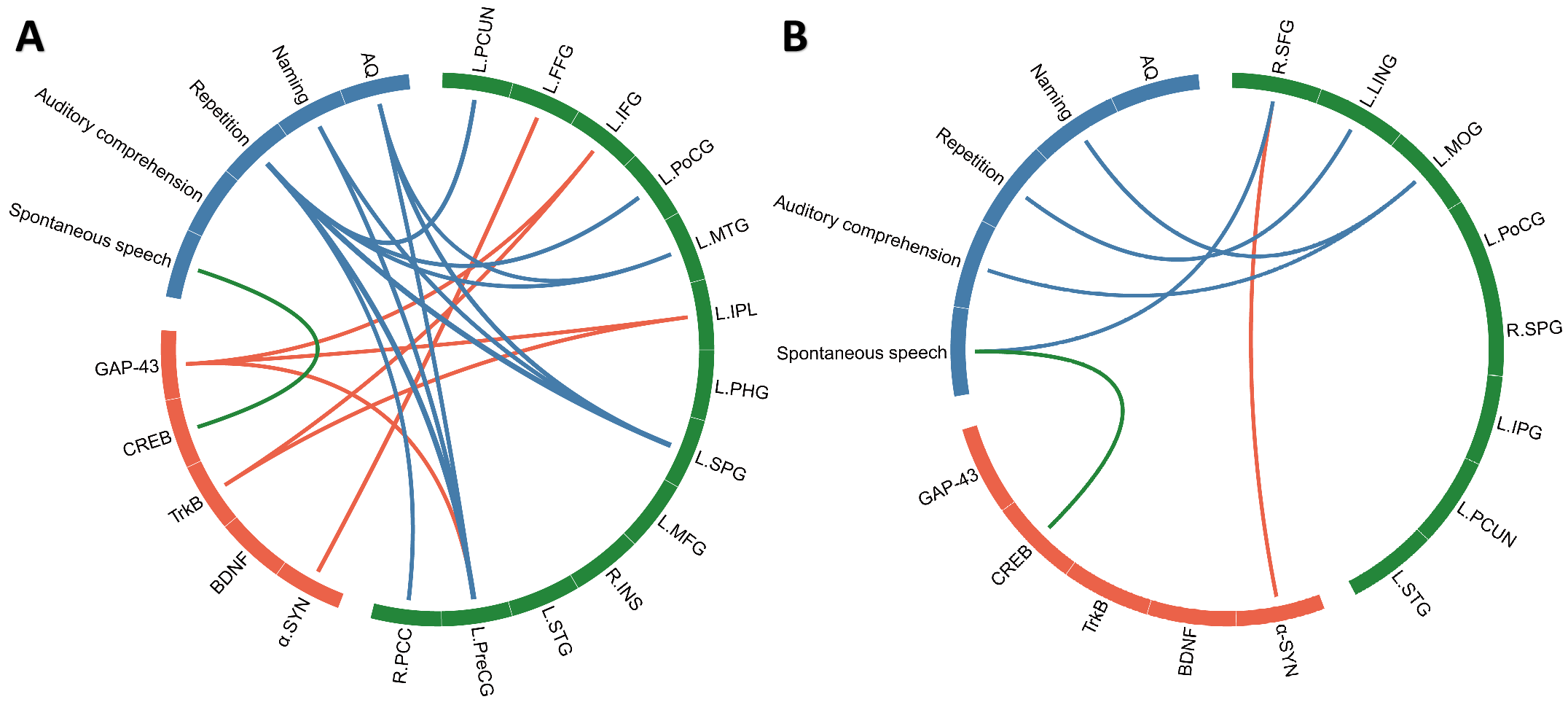


**Figure S1.** Correlation Networks of structural brain characteristics, synaptic-associated proteins and language assessments. A. Correlation network of SBM indices (CT), synaptic-associated proteins and language assessments. B. Correlation network of VBM indices (GMV), synaptic-associated proteins and language assessments. Green block represents brain regions identified by SBM and VBM analyses; orange block represents synaptic-associated proteins; blue block represents language function.
